# Movement behaviours and anxiety symptoms in Chinese college students: A compositional data analysis

**DOI:** 10.3389/fpsyg.2022.952728

**Published:** 2022-09-15

**Authors:** Luomeng Chao, Rui Ma, Weiwei Jiang

**Affiliations:** ^1^Physical Education Teaching Unit, Inner Mongolia Medical University, Hohhot, China; ^2^Postdoctoral Research Station in Public Administration, Zhengzhou University, Zhengzhou, China; ^3^School of Physical Education (Based School), Zhengzhou University, Zhengzhou, China; ^4^Centre for Mental Health, Shenzhen University, Shenzhen, China

**Keywords:** anxiety, sleep, sedentary behaviour, moderate to vigorous physical activity, young adults

## Abstract

In the current research, sleep duration, sedentary behaviour, physical activity, and their relationship with several anxiety symptoms among college students were examined. This study was a cross-sectional study, and study respondents were recruited from college students. A total of 1,475 of college students were included for analysis. Sedentary behaviours and physical activity were assessed by the International Physical Activity Questionnaire Short Form, while sleep duration was assessed by the Chinese version Pittsburgh Sleep Quality Index. To assess the anxiety symptoms of study respondents, the Generalised Anxiety Disorder-7 was used. The compositional data was analysed in R to estimate the associations between replacements among physical activities, sedentary behaviours, and sleep and anxiety symptoms. Results indicated a greater impact was imposed on the alleviation of anxiety symptoms by substituting sedentary behaviours with physical activity at moderate to vigorous intensity. In the current research, the significance of moderate to vigorous physical activity should be highlighted in preventing anxiety among Chinese college students.

## Introduction

Since the outbreak of COVID-19, people’s life has been affected from various aspects around the world (Pokhrel and Chhetri, 2021). In the meanwhile, many measures have been taken by individuals to avoid contracting the disease, such as working from home, quarantine, and going out less frequently (Lim et al., 2020). Consequently, people’s lifestyles have changed. For example, people’s sleep habits have been disturbed. Such lifestyle changes have further led to mental health issues in different populations, such as college students. During the pandemic period, universities all over the world mostly closed their campuses and turned to provide online teaching for their students (Yıldırım et al., 2021). These measures may have changed students’ living and studying patterns to a certain extent, resulting in mental health problems, such as depression or anxiety among college students (Rajab et al., 2020). Hence, the effect of the COVID-19 on individual’s mental health has become a great concern for higher education students all over the world.

As defined by previous research, movement behaviour refers to various activities that individuals engage in their daily lives (Chen and Yan, 2020; Shen et al., 2020). This includes physical activity and sedentary behaviours (Mañas et al., 2018; Huang et al., 2021; Chen et al., 2022). Sleep is also a component of movement behaviour that has a significant impact on people’s physical and mental health (Chen and Yan, 2020; Shen et al., 2020; Liu et al., 2022). Studies have shown that changes in movement behaviours would result in mental health issues of college students (Savage et al., 2020). Many researchers have found that with increasing sedentary behaviours hours, college students tend to experience a rising level of anxiety (Lee and Kim, 2019; Chen and Yan, 2020; Shen et al., 2020; Liu et al., 2022). Jiang et al. (2020) also reached a similar conclusion that sedentary behaviours was positively associated with anxiety and even suicidal attempts among first-year college students based on one empirical study conducted in Chinese college students. Besides, researchers also investigated the associations between sedentary behaviours, physical activity with mental health issues. Yasunaga et al. (2018) conducted a study using cross-sectional analysis and suggested that light physical activity, as the replacement of sedentary behaviours with other components of movement behaviours, might result in a lower level of depression. Likewise, Pengpid and Peltzer (2019) studied the relationship between sedentary behaviours and physical activity with mental health issues. This study found a negative association between the duration of sedentary behaviours and physical activities and symptoms of depression and/or anxiety, researchers also further examined and compared the effects of moderate to vigorous physical activity and light physical activity on these mental health issues, respectively (Shi et al., 2022). Moreover, previous research has indicated that changes in sleep were significantly associated with individuals’ mental health. In a nationwide study conducted in Japan, researchers recruited 99,668 participants to complete a questionnaire regarding their lifestyle and mental health status (Kaneita et al., 2007). The results demonstrated that there was a U-shape association between individuals’ average daily sleep time and mental health. Individuals who have 7–9 h of sleep appear to have the healthiest mental health status, while people who sleep less than 7 hours or more than 9 hours a day have lower scores on the mental health assessment. According to a meta-analysis conducted by Baglioni et al. (2016), sleep deprivation was significantly associated with mental illnesses such as depressive and general anxiety disorder. Therefore, it is significant to take sleep into account when investigating individuals’ behavioural factors associate with anxiety symptoms.

Nevertheless, when examining the association between physical activity and mental health status among Chinese college students, the results appear to be mixed and unclear. A cross-sectional study gathered data from 1,846 Chinese undergraduates, and it demonstrated that individuals who engaged in higher levels of physical activity tend to have lower risks of experiencing anxiety symptoms, which indicated a negative relationship between individuals’ physical activities and anxiety levels (Gupta et al., 2020). On the contrary, a recent study conducted by Feng et al. (2022) investigated the relationship between physical activity and posttraumatic stress disorder during the COVID-19 pandemic. The results suggested that individuals with higher physical activity levels appear to have lower odds of experiencing posttraumatic stress disorder symptoms after the pandemic, which is contrary to the researchers’ hypothesis and the findings of many previous studies. As a result of those mixed findings, it is significant to further investigate the relationship between physical activity and mental health status among the Chinese undergraduate population.

Apart from the numerous studies conducted to examine the associations between sedentary behaviours, physical activities, sleep, and mental health including depression and anxiety, a novel statistical analysis approach, compositional data analysis, has become popular in such research (Bu et al., 2021). Individuals’ lifestyle behaviours can be influenced with different components of movement behaviours, implying that time spent in a certain activity (e.g., sedentary behaviours) is closely intertwined with time spent on other activities. Hence, the compositional data analysis has been commonly applied by researchers to explore the relationship between the intertwined components of movement behaviours and individuals’ mental health, given the compositional nature of time allocation among movement behaviours (Gupta et al., 2018). Kitano et al. (2020) have illustrated a significant association between the time distribution of 24-hour movement behaviours and mental health among workers. Also, Kitano et al. (2020) also investigated the effects of reallocating different movement behaviours, clearly demonstrating relationships between activity substitutions and mental health. As the COVID-19 epidemic is ongoing, an increasing number of studies have embarked on delving into the relationships between individuals’ movement behaviours and mental health during the epidemic, relying on personal lifestyle changes, such as increased sedentary behaviours or sleep with decreased physical activity. For example, the newly released research implemented by Brusaca et al. (2021) discussed the change in people’s lifestyles and investigates the relationship between movement behaviour change and mental health issues such as stress and anxiety by using the compositional data analysis approach. Another study conducted by Chong et al. (2021) also suggested the distinct relationship between different movement behaviours including sleep and psychological distress with a compositional data analysis method. The use of the compositional data analysis technique can provide an efficient reference for the public to allocate their time to activities contributing to mental health.

During the period of COVID-19, it is essential to utilise the compositional data analysis approach to delve into the relationship between components of movement behaviours and anxiety symptoms among college students, considering the reallocations of sleep, sedentary behaviours, and physical activity. This can give recommendations to college students so that their mental health can be improved.

## Materials and methods

### Study participants

This present study was a cross-sectional study. In this study, an online survey network was used to recruit the participants. Firstly, study participants were determined based on a convenient sampling method. Then, study participants who agreed to participate in research were sent a survey link, and they were required to complete the online questionnaire about approving the recruitment and data collection process of research participants as well as the details of the research protocol. Before filling out the questionnaire, study participants offered online consent, only those who have provided informed consent can be eligible to participate. In total, 1,942 college students took part in this survey, 1,846 of them provided responses, with a response rate of 95.1%. Finally, 1,475 college students were included, since they offered valid and complete data for analysis.

### Measures

#### Sedentary behaviour and physical activity

To assess sedentary behaviours and physical activity, the International Physical Activity Questionnaire Short Form (IPAQ-SF) was used. Moreover, study respondents were instructed to report their walking (light physical activity), moderate to vigorous physical activity and time spent sitting (sedentary behaviours) in the last week. According to the previous research, IPAQ-SF displays good validity and reliability in Chinese adults (Qi et al., 2020). In details, as for the IPAQ-SF, there is a high intraclass correlation coefficient (ICC), which is 0.97 (95% confidence interval [CI]: 0.95, 0.98) for SB, and an ICC of 0.85 (95% CI: 0.75, 0.91) for moderate physical activity, and 0.75 (95% CI: 0.60, 0.85) for vigorous PA. The IPAQ-SF is a reliable assessment for estimation of sedentary behaviours and physical activity in Chinese adults (Yıldırım et al., 2021). As for the validity, according to the prior study, IPAQ-SF displays sound validity against a device-based measure (Maher et al., 2018; Kurth and Klenosky, 2021).

#### Sleep duration at night

Questions are designed by referring to the Chinese version of the Pittsburgh Sleep Quality Index (PSQI) to assess sleep duration, that is, “how long did you sleep at night during the past 30 days?” (Chung et al., 2009). The item of measure displays sound reliability and validity in Chinese adults (Wu et al., 2019; Chi et al., 2021).

#### Anxiety symptoms

Anxiety symptoms of study participants was assessed by the 20-item self-report questionnaire, that is the Zung Self-Rating Anxiety Scale (Z-SAS) (Dunstan and Scott, 2020). The Z-SAS includes measures of state and trait anxiety based on scoring in four groups of manifestations: cognitive, autonomic, motor, and central nervous system symptoms. Responses to each item range from 1 (a little of the time) to 4 (most of the time) with higher scores indicating more severe anxiety. Standardised scores of the Z-SAS were used in the statistical analysis of our study.

#### Sociodemographic

Sociodemographic variables contain weight (in kilogramme, for BMI, that is, body mass index), perceived family affluence (scaled 0–10), self-reported height (cm), dwelling place (urban or rural), age, gender, and siblings (yes or no). For the statistical model, those variables were considered as covariates.

### Statistical analysis

Using compositions (version 1.40-1) (Van den Boogaart and Tolosana-Delgado, 2008) and RGB compositions (version 0.92-7), R, and latest (version 0.9-35) (Templ et al., 2011) packages, the analysis of compositional data has been performed in R^1^ (R Core Team, version 3.6.1, 2019). For compositional and standard descriptive statistics, the comparison is performed by calculating them. The compositional means have been obtained through the calculation of the geometric mean of every behaviour (time spent on moderate to vigorous physical activity, light physical activity, sedentary behaviours and sleep), and the data has been normalised to the identical constant. The measure is identical to the relative and symmetrical scale of data (Aitchison, 1982). In the current research, a compositional approach based on an isometric log-ratio (ILR) data transformation has been adopted, and such an approach has been adapted from Hron (Hron et al., 2012) to adjust the models for time spent on other behaviours. To sum up, a sequential binary partition process has been adopted to generate the ILR coordinates (Egozcue and Pawlowsky-Glahn, 2005), and it was obtained by composition partitioning because one set was designated to appear in the first ILR coordinate numerator, while the other in the denominator. Moreover, one set has been separated into two sets, while the parts have been coded to be in the uninvolved parts (0), the denominator (–1), and numerator (+1) respectively. The final ILR is the geometric mean of normalised log ratios of the parts (Dumuid et al., 2019). In addition, apart from that, covariates have been considered as the explanatory variables. To not violate the assumption, the multiple linear regression models of ILR have been examined for linearity, homoscedasticity, normality and outliers. Moreover, the significance of the composition of physical behaviours has been measured with the function of ‘car: Anova’, in which Wald Chi-squared has been adopted to conduct Type II tests. By the following marginality as a principle, every covariate has been examined. In addition, the models of ILR multiple linear regression have been adopted to check the difference in outcome variable associated with the reallocation of a fixed period between different physical actions, while the 3rd and 4th remain unchanged. Five minutes of reallocation between different pairs of physical behaviours can be simulated through the systematic creation of specific new composition of activity, with the mean composition of the sample as the baseline, or composition start. As for new compositions, they have been demoted as coordinate sets of ILR, and each value has been subtracted from average composition ILR coordinates to generate the ILR differences. Therefore, *ilr* differences have been adopted in the linear models so that the estimated difference can be determined (95% CI). Meanwhile, it is performed once again for pairwise reallocation, with 5 minutes as an increment (5–15 minutes) respectively. The reallocation rationale at 5 minutes is based on the revised version of PA guidelines in 2019 for the United Kingdom (Smith et al., 2021) and the United States (Bezerra et al., 2021), and the 10-minute minimum has been removed concerning the duration of all age groups, as a result of evidence insufficiency. All statistical analyses were conducted at a significance level of *p* < 0.05.

## Results

A total of 1,475 study participants with a mean age of 20.7 years old were included in the final analysis (Table 1). Of them, 68% were females and 33.8% had siblings. 68.9% of study participants lived in urban areas. The mean of perceived family affluence was 5.70 with a standard deviation of 1.63. The study sample spent 50% of the day (24 hours) in sleep, 39.77% in sedentary behaviour, 5.60% in light physical activity, and 4.63% in moderate to vigorous physical activity. Lastly, the mean score of anxiety symptoms was 41.79 with a standard deviation of 9.82. The mean body mass index of study participants was 20.13 (standard deviation: 2.82).

**TABLE 1:** Participant characteristics.

Characteristics (*n* = 1475)	
Age, mean (SD), year	20.7 (1.60)
Female, *n* (%)	1,003 (68.0)
With siblings, *n* (%)	498 (33.8)
Residence (urban), *n* (%)	1,016 (68.9)
Perceived family affluence, mean (SD)	5.70 (1.63)
Time use^a^, *n* (%), min/d	
Sleep	720.03 (50)
Sedentary	572.69 (39.77)
LPA	80.61 (5.60)
MVPA	66.67 (4.63)
Body mass index, mean (SD)	20.13 (2.82)
Anxiety symptoms scores, mean (SD)	41.79 (9.82)

^a^Time-use composition is presented as geometric means, adjusted to a sum of 1,440 minutes and 100%. LPA, light physical activity; MVPA, moderate to vigorous physical activity.

When data were considered as a composition, adjusted for all the sociodemographic variables; the components of movement behaviours was significantly associated (95% CI) with anxiety symptoms scores (*p* < 0.0001). The results based on temporal substitution can be found at Table 2, we found that increases in moderate to vigorous physical activity, light physical activity, or Sleep, at the expense of sedentary behaviours, was associated with a negative change in anxiety symptoms scores. Moreover, the addition of moderate to vigorous physical activity conferred the greatest decrease in anxiety symptoms scores, whilst the addition of sleep or light physical activity yielded improvements of roughly same magnitude. Specifically, we found that anxiety symptoms scores decreased significantly from adding MVPA (−0.10, 95%CI: −0.16, −0.04), light physical activity (−0.05, 95%CI: −0.08, −0.02), or sleep (−0.00, 95%CI: 0.00, 0.01) at the expense of SB at 5 minutes, ranging to −0.22 (95%CI: −0.36, −0.09), −0.12 (−0.20, −0.04), and 0.00 (−0.01, 0.02) for moderate to vigorous physical activity, light physical activity, and sleep, respectively, at 15 minutes.

**TABLE 2:** Estimated differences in anxiety symptoms scores changes among reallocations sleep, sedentary behaviour and physical activity.

		Prediction	95% CI		*p*
**5 min**					
Sleep	Sedentary	0.00	0.00	0.01	1.0000
Sleep	LPA	0.05	0.02	0.09	0.0051
Sleep	MVPA	0.16	0.06	0.26	0.0018
Sedentary	Sleep	0.00	–0.01	0.00	1.0000
Sedentary	LPA	0.05	0.02	0.09	0.0051
Sedentary	MVPA	0.16	0.06	0.26	0.0018
LPA	Sleep	–0.05	–0.08	–0.02	0.0011
LPA	Sedentary	–0.05	–0.08	–0.02	0.0011
LPA	MVPA	0.11	0.01	0.22	0.0397
MVPA	Sleep	–0.10	–0.16	–0.04	0.0011
MVPA	Sedentary	–0.10	–0.16	–0.04	0.0011
MVPA	LPA	–0.04	–0.12	0.03	0.3000
**10 min**					
Sleep	Sedentary	0.00	–0.01	0.01	1.0000
Sleep	LPA	0.12	0.04	0.20	0.0033
Sleep	MVPA	0.70	0.28	1.12	0.0011
Sedentary	Sleep	0.00	–0.01	0.01	1.0000
Sedentary	LPA	0.12	0.04	0.20	0.0033
Sedentary	MVPA	0.70	0.28	1.12	0.0011
LPA	Sleep	–0.09	–0.14	–0.03	0.0014
LPA	Sedentary	–0.09	–0.14	–0.03	0.0014
LPA	MVPA	0.61	0.17	1.05	0.0066
MVPA	Sleep	–0.17	–0.27	–0.07	0.0009
MVPA	Sedentary	–0.17	–0.27	–0.07	0.0009
MVPA	LPA	–0.05	–0.19	0.09	0.4937
**15 min**					
Sleep	Sedentary	0.00	–0.01	0.02	1.0000
Sleep	LPA	0.20	0.07	0.33	0.0026
Sleep	MVPA	–0.78	–1.25	–0.30	0.0013
Sedentary	Sleep	0.00	–0.02	0.01	1.0000
Sedentary	LPA	0.20	0.07	0.33	0.0026
Sedentary	MVPA	–0.78	–1.26	–0.30	0.0015
LPA	Sleep	–0.12	–0.20	–0.04	0.0033
LPA	Sedentary	–0.12	–0.20	–0.04	0.0033
LPA	MVPA	–0.93	–1.41	–0.45	0.0002
MVPA	Sleep	–0.23	–0.36	–0.09	0.0009
MVPA	Sedentary	–0.22	–0.36	–0.09	0.0015
MVPA	LPA	–0.03	–0.24	0.18	0.7919

Min, minutes; LPA, light physical activity; MVPA, moderate to vigorous physical activity; CI, confidence interval.

## Discussion

This research aimed at investigating the relationship between reallocation of time-use behaviours and anxiety symptoms during the pandemic period in Chinese college students, providing evidence-base for public health interventions aimed at preventing anxiety. In general, this research found that the redistribution of the 24-hour time-use component was related to anxiety symptoms in Chinese college students during the pandemic period. However, this study primarily illustrated that either the increased sleep replaced from reduced sedentary behaviours was not associated with anxiety symptoms scores. In spite of this, other replacements have shown their relationship with anxiety scores respectively. In particular, the reduction of light physical activity from other sedentary behaviours was positively related to higher anxiety scores (regardless of the duration of the replacement). Besides, adding moderate to vigorous physical activity from removing sleep, sedentary, or light physical activity can consistently lead to a lower anxiety score. A detailed discussion of the results is presented below.

The results obtained in this study highlighted the positive effects of moderate to vigorous physical activity on preventing anxiety in college students, since increased moderate to vigorous physical activity together with reduced SB can contribute to decreased anxiety symptom. Meanwhile, substantial evidence has also explained the positive effect of moderate to vigorous physical activity on anxiety, regardless of age, gender, education level and other confounders. Importantly, Carter et al. (2021) concluded that physical activity can be an effective approach to reduce anxiety symptoms among children and adolescents. Other earlier studies have also acknowledged the health benefits of physical activity in addressing anxiety disorder (Penedo and Dahn, 2005; McCurley et al., 2017; de Oliveira et al., 2019). Ströhle (2009) further suggested that regular physical activity can decrease symptoms of anxiety. As breaking out COVID-19, irregular sleep or sedentary lifestyles have become more popular, triggering more people tend to stay at home. This probably further contributes to individual emotional problems such as anxiety disorder. Hence, when people participate in moderate to vigorous physical activity, their anxiety symptoms may decrease to a certain extent. In this research, increasing moderate to vigorous physical activity while removing sedentary behaviours at 5, 10, or 15 minutes presented a negative relationship with anxiety symptoms, which confirmed the negative association between moderate to vigorous physical activity and anxiety. This finding concurs with previous studies using novel research methods. For example, Strong et al. (2005) demonstrated that increased level of moderate to vigorous physical activity could facilitate mental health and prevent anxiety problems in adolescents. Beyond that, based on the cross-sectional analysis, one empirical study conducted by McCurley et al. (2017) suggested a relationship between moderate to vigorous physical activity and fewer anxiety symptoms and status among the elderly. However, it should be noted that these studies were conducted before the COVID-19 outbreak. Nevertheless, there have been studies investigating the relationship between moderate to vigorous physical activity and anxiety symptoms, which also display similar results. A cross-sectional research carried out by Schuch et al. (2020) in Brazil indicated that there was less possibility for people participating in moderate to vigorous physical activity to show anxiety symptoms. Additionally, an investigation conducted by McCormack et al. (2020) concentrated on the relationship between physical activity and anxiety among American children during the COVID-19 outbreak. Their findings displayed a negative relationship between moderate to vigorous physical activityand anxiety symptoms, implying that frequent participation in moderate to vigorous physical activity could greatly reduce or even prevent anxiety symptoms among young people. Therefore, this study could serve as additional evidence to the literature that moderate to vigorous physical activity would be good for reducing anxiety symptoms and should be useful in promoting policies on related issues, such as encouraging people to participate in moderate to vigorous physical activity.

Another result worthy discussing in this research concerns replacing sleep or sedentary behaviours with light physical activity and its associated effects on anxiety symptoms. As shown by the results based on 5, 10, or 15 minutes reallocations, the estimated differences in anxiety symptoms scores appear to be similar when sleep or sedentary behaviours was substituted by light physical activity. More specifically, replacing either sleep or sedentary behaviours with light physical activity generally reduced anxiety symptoms in this research, which suggests that the substitution of sleep or sedentary behaviours with light physical activity would probably prevent anxiety. By contrast, some earlier studies displayed results different from our study. According to the research by Fanning et al. (2017), it does not witness significant effects of replacing sedentary behaviours with light physical activity, which contradicts our results. Although certain previous studies have not reached a similar conclusion to this study, different studies illustrated the relationship between adding light physical activity with removing sleep or sedentary and anxiety symptoms. In a cross-sectional study conducted by Helgadóttir et al. (2015), the authors suggested that individuals suffering from anxiety disorders should be encouraged to reduce sedentary time and add more light physical activity. Moreover, contrary to the findings achieved by Fanning et al. (2017) discussed above, Dillon et al. (2018) have concluded a significant decline in anxiety symptoms by substituting light physical activity for sedentary behaviours among the middle-aged. Even though the results of the study conducted by Fanning et al. (2017) and the research by Dillon et al. (2018) are different, it should be highlighted that Fanning et al. (2017) concentrated on the relationship among the elderly while Dillon et al. (2018) surveyed the middle-aged people. Hence, age is probably a factor influencing the association between.

A point that should be illustrated in this research refers to the differences in reduced sedentary behaviours between increased light physical activity and increased moderate to vigorous physical activity. According to the results, increased moderate to vigorous physical activity has a more significant effect on lowering anxiety symptoms than increased light physical activity does with declined sedentary behaviours. In detail, when it comes to differences in anxiety scores, moderate to vigorous physical activity can be almost twice as effective as light physical activity. However, this finding was somewhat consistent with most studies, yet the other half has called for a debate. On the one hand, previous research indicated that replacing sedentary behaviours with moderate to vigorous physical activity could reduce or prevent anxiety symptoms (Meyer et al., 2020; Tully et al., 2020; Kandola et al., 2021). On the other hand, several studies had doubt on the impact of substituting sedentary behaviours for light physical activity. A research conducted by Kandola et al. (2021), the researchers suggested that replacing sedentary behaviours with light physical activity could not only decrease depressive symptoms but might necessarily decrease anxiety symptoms. Nevertheless, researchers have not completely denied the effects of replacing sedentary behaviours with light physical activity and assert that further studies are needed to examine. One study conducted by Tully et al. (2020) found that replacing sedentary behaviours with light physical activity or moderate to vigorous physical activity could decline anxiety symptoms among older people, in support of the results in this study even if time duration in the two studies were different. Furthermore, the study carried out by Meyer et al. (2020) illustrated that both replacing sedentary behaviours with either light physical activity or moderate to vigorous physical activity could reduce anxiety symptoms in young adults.

There are several limitations carried by the current study. Firstly, the data of this present research was collected by implementing a cross-sectional method. Although significant correlations were found between the variables, the data should be interpreted with caution and causal relationships cannot be concluded from this study. Also, a third variable cannot be eliminated from the positive correlation between physical activity and participants’ lower anxiety scores. For instance, a past study has demonstrated that individuals’ socio-economical status would contribute to their higher likelihood of adopting a healthy lifestyle, while the socio-economical status background also acts as a protective factor for people’s mental health status. Future studies should be conducted to further examine the relationship between physical activity and anxiety symptoms by taking the socio-economical status factor into account.

Moreover, the current study only recruited participants from the Chinese college student in southern China, and the findings may not be generalisable to populations living in other regions. Also, the current study used self-reported questionnaires to collect data, which is subject to respondents’ bias and social desirability. Last, cross-sectional study design fails to allow us to determine cause-and-effect inference. Despite the limitations, the findings of the current study have significant implications. In general, as anxiety disorders are prevalent in Chinese college students and can lead to problematic behaviours, it is important to explore potential solutions to reduce the symptoms. Based on the current study, promoting health literacy and an active lifestyle might be helpful to prevent anxiety disorder in Chinese college students, as research has indicated that the promotion of an active lifestyle is positively associated with fewer mental health problems. Potential practices that have a positive impact on individuals’ physical activity might also be effective to reduce anxiety symptoms of Chinese college students. Future studies are needed to further explore the effectiveness of these planned actions.

## Conclusion

This study offered evidence concerning favourable replacements among sleep, sedentary behaviour, light physical activity, and moderate to vigorous physical activity were associated with anxiety symptoms among Chinese college students. Replacing more sedentary behaviour with moderate to vigorous physical activity might result in greater effects on reducing anxiety symptoms. Future studies are encouraged to adopt improved study designs for confirmation or negation of our research findings.

## Data availability statement

The original contributions presented in this study are included in the article/supplementary material, further inquiries can be directed to the corresponding author.

## Ethics statement

Written informed consent was obtained from the individual(s) for the publication of any potentially identifiable images or data included in this article.

## Author contributions

LC: writing—original draft. RM: formal analysis. LC and RM: writing—review and editing. WJ: data curation. All authors contributed to the article and approved the submitted version.

## Footnotes

1
http://cran.r-project.org
